# Increased Anion Channel Activity Is an Unavoidable Event in Ozone-Induced Programmed Cell Death

**DOI:** 10.1371/journal.pone.0013373

**Published:** 2010-10-13

**Authors:** Takashi Kadono, Daniel Tran, Rafik Errakhi, Takuya Hiramatsu, Patrice Meimoun, Joël Briand, Mari Iwaya-Inoue, Tomonori Kawano, François Bouteau

**Affiliations:** 1 Laboratoire d'Electrophysiologie des Membranes, Université Paris Diderot-Paris 7, Institut de Biologie des Plantes, Bât 630, Orsay, France; 2 Faculty of Agriculture, Kyushu University, Hakozaki, Higashi-ku, Fukuoka, Japan; 3 Graduate School of Environmental Engineering, University of Kitakyushu 1-1, Hibikino, Wakamatsu-ku, Kitakyushu, Japan; University of Melbourne, Australia

## Abstract

**Background:**

Ozone is a major secondary air pollutant often reaching high concentrations in urban areas under strong daylight, high temperature and stagnant high-pressure systems. Ozone in the troposphere is a pollutant that is harmful to the plant.

**Principal Findings:**

By exposing cells to a strong pulse of ozonized air, an acute cell death was observed in suspension cells of *Arabidopsis thaliana* used as a model. We demonstrated that O_3_ treatment induced the activation of a plasma membrane anion channel that is an early prerequisite of O_3_-induced cell death in *A. thaliana.* Our data further suggest interplay of anion channel activation with well known plant responses to O_3_, Ca^2+^ influx and NADPH-oxidase generated reactive oxygen species (ROS) in mediating the oxidative cell death. This interplay might be fuelled by several mechanisms in addition to the direct ROS generation by O_3_; namely, H_2_O_2_ generation by salicylic and abscisic acids. Anion channel activation was also shown to promote the accumulation of transcripts encoding vacuolar processing enzymes, a family of proteases previously reported to contribute to the disruption of vacuole integrity observed during programmed cell death.

**Significance:**

Collectively, our data indicate that anion efflux is an early key component of morphological and biochemical events leading to O_3_-induced programmed cell death. Because ion channels and more specifically anion channels assume a crucial position in cells, an understanding about the underlying role(s) for ion channels in the signalling pathway leading to programmed cell death is a subject that warrants future investigation.

## Introduction

Ozone produced by a complex series of photochemical reactions from primary precursor emissions of nitrogen oxide and volatile organic compounds, is a major secondary air pollutant. Chronic exposures to low O_3_ concentrations have a negative impact on crop yields by reducing photosynthesis and growth, and inducing premature leaf senescence in sensitive plants [1]. Acute transient O_3_ exposures cause cell death, visible as O_3_ lesions in the leaves [2] that resemble a programmed cell death (PCD) associated with the hypersensitive response (HR) in plant-pathogen interactions [3], [4], [5]. The primary site of O_3_ interaction within plant cells is the apoplast where O_3_ challenges the antioxidant protection of the cell. Accordingly, O_3_ sensitivity generally correlates with the ascorbate status of the apoplast, which is an important signal initiation point [6]. Ozone also breaks down into various reactive oxygen species (ROS) namely hydrogen peroxide, singlet oxygen and hydroxyl radicals [6]. A quick burst of ROS produced by plant cells is also induced by high concentrations of O_3_ [3] and it resembles the oxidative burst of the HR in incompatible plant–pathogen interactions [7]. In plants, an exposure to O_3_, also leads to a rapid increase in cytosolic free calcium ([Ca^2+^]_cyt_) [8], [9], [10]. This increase in cytosolic Ca^2+^ concentration is sensitive to Ca^2+^ chelators, ion channel blockers, and ROS scavengers thus suggesting that calcium influx from the apoplast acts as a secondary messenger initiating oxidative cell death [4], [10] in addition to rapid changes in the protein phosphorylation pattern [11]. The production of ROS finally leads to a PCD characterized by the early release of cytochrome c from mitochondria, activation of proteases, DNA fragmentation, electrolyte leakage and ultrastructural changes characteristic of PCD [3], [4], [5], [10]. Responses that follow the O_3_ challenge also include changes in gene expression and protein synthesis, and an accumulation of plant stress hormones, ethylene, salicylic acid (SA), abscisic acid (ABA) and jasmonic acid (JA) [4], [12], [13], [14], [15], [16], that regulate the induction and spreading of the oxidative stress symptoms [3], [14], [17], [18]. Treatment with O_3_ also induces a rapid accumulation of NO, which could coincide with the formation of HR-like lesions, suggesting that NO is also an important signalling molecule in the plant response to O_3_ [19].

Anion channels play fundamental roles in key biological processes including plant cell response to environmental stresses [20], [21], [22]. Several types of anion channel differing in their voltage dependence, kinetic properties and anion selectivity have been characterized, mostly by electrophysiological techniques. R-type anion channel activation is an essential step of the ROS-dependent innate immune response in Arabidopsis suspension cells [23]. In the same model system, ROS generation and change in [Ca^2+^]_cyt_ participate to ABA-induced anion channel regulation [24], [25], [26]. Recently, an anion channel SLAC1 [27], [28], [29], [30], [31] was shown to be essential for stomatal closure in response to ozone, Ca^2+^ ions and H_2_O_2_ in Arabidopsis [31], suggesting that anion efflux could be induced in guard cells by O_3_ through an increase in anion channel activity. Anion effluxes are also amongst the earliest responses observed in plant cells following recognition of pathogenic signals. For instance, cryptogein induces a rapid and massive activation of anion channel mediated nitrate efflux regulated by Ca^2+^-dependent events [32], [33]. This NO_3_^−^ efflux was shown to be necessary for the mediation of the cryptogein-induced oxidative burst, the induction of defence-related genes and the development of the HR. In this model, NO_3_^−^ efflux was also shown to promote the accumulation of transcripts encoding vacuolar processing enzymes (VPEs) [33], a family of proteases showing caspase-1 activity [34] and reported to contribute to the disruption of vacuole integrity observed during the HR [35]. Anion channel regulation was also shown to be an essential component of harpin- and oxalic acid-induced plant PCD [36], [37]. The involvement of anion channels as a critical component of the cell death process in plants is similar to their key role in animal apoptosis. Indeed, several studies have reported that in various types of mammalian cells, the activation of a plasma membrane Cl^−^ channel is an early prerequisite to apoptotic events including cell shrinkage (termed AVD for apoptotic volume decrease), cytochrome c release, the activation of proteases (including caspases) and nucleases and, ultimately, cell death [38], [39]. Taken together, these data suggest that anion channel activation is not a passive secondary feature of plant responses to stress, but a driver of these processes.

Recent research has shown that the similarity of O_3_-induced cell death and hypersensitive cell death is not only external [2], [40] since these phenomena also share many physiological and molecular features [3], [11]. Therefore, we have analyzed the role of anion channels in O_3_-induced cell death signalling pathways using *Arabidopsis thaliana* cells as a model. It was found that an O_3_ challenge induced the activation of a plasma membrane anion channel which was an early prerequisite of O_3_-induced cell death in *A. thaliana*. This oxidative cell death was mediated by the interplay of anion channel activation, Ca^2+^ influx, ROS generation and included an increase in VPE transcripts. Furthermore, ABA and salicylic acid appear to participate to this anion channel activation leading to O_3_-induced cell death.

## Results

### O_3_-induced cell death in *A. thaliana* suspension-cultured cells

**Figure 1:**
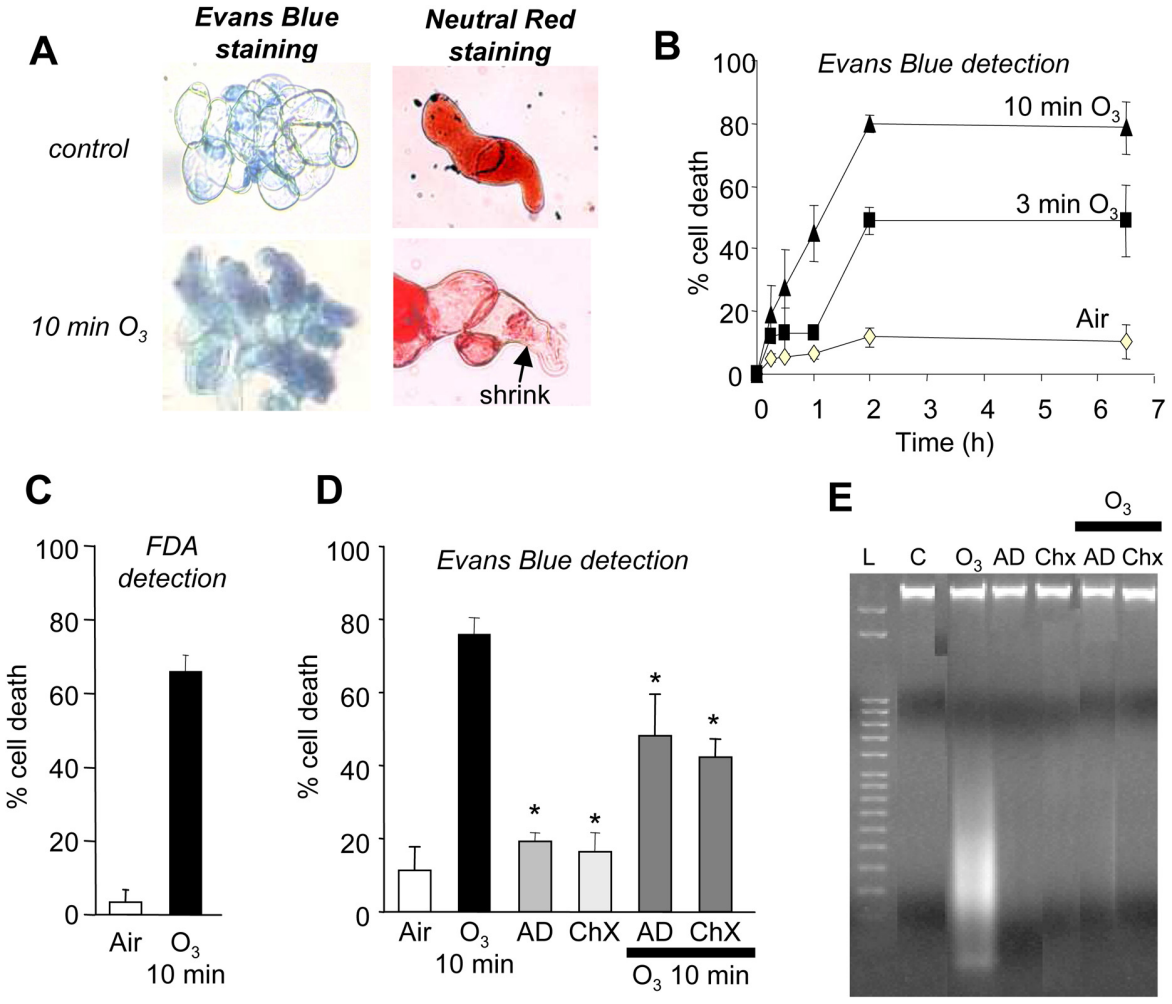
O_3_-induced cell death in *A. thaliana* suspension cultured cells. **A.** Light micrographs of ozone-treated *A. thaliana* cells stained with Evans Blue or Neutral Red. Cells were exposed 10 min to ozonized air and incubated for a further 6.5 h for development of cell death. **B.** Time-dependent development of O_3_-induced cell death in *A. thaliana* cells. Cell death was induced by exposing the cell suspensions to a pulse of ozonized air lasting for either 3 or 10 min. Controls correspond to a 10 min pulse with air only. O_3_-treated and pretreated cell suspensions (0.2 ml) were sampled and transferred to a 1.5 ml tube at the end of the O_3_ exposure. For each treatment, cells were incubated for up to 6.5 h with samples taken at 0, 0.25, 0.5, 1, 2 and 6.5 h, for cell staining with Evans blue and subsequent counting. **C.** Cell death extent detected by the FDA technique after 6 h. **D.** Effect of pretreatment with actinomycin D (AD, 20 µg/ml) or cycloheximide (Chx, 20 µg/ml) on ozone-induced cell death detected by Evans blue staining. The data correspond to means of at least 4 independent replicates and error bars correspond to SE. **E.** Fragmentation of nuclear DNA detected by gel electrophoresis after a 10 min exposure to ozonized air with or without either actinomycin D (20 µg/ml) or cycloheximide (20 µg/ml). Representative results from three independent experiments are shown. DNA molecular weight markers (bp) are shown on the left (lane L).

We first checked if O_3_ induced cell death in our model plant system by exposing Arabidopsis cell cultures to a pulse of ozonized air. Vacuole shrinkage was observed which led to completely collapsed cells and finally cell death as determined by Evans blue or neutral red staining (Figure 1A). The percentage of dead cells reached a plateau about 2 h after O_3_ treatment (Figure 1B), the degree of cell death being dependent on O_3_ treatment duration, with about 50% and 80% of dead cells detected 2 h after a 3 min and a 10 min O_3_ treatment, respectively (Figure 1B). The number of dead cells was similar when quantified by the fluorescein diacetate (FDA) spectrofluorimetric method (Figure 1C). In order to check whether this O_3_-induced cell death was due to an active mechanism requiring active gene expression and cellular metabolism, A. thaliana cell suspensions were treated with actinomycin D (AD), an inhibitor of RNA synthesis, or with cycloheximide (Chx), an inhibitor of protein synthesis, at 20 µg.mL^−1^ each, 15 min prior to O_3_ exposure. Actinomycin D and Chx significantly reduced the O_3_-induced cell death (Figure 1D). These results indicated that this cell death required active cell metabolism, namely gene transcription and *de novo* protein synthesis. Fragmentation of nuclear DNA was observed by agarose gel analysis of DNA extracted from cell suspensions after a 10 min treatment with O_3_ (Figure 1E). This DNA fragmentation was also dependent on active gene expression and *de novo* protein synthesis since it was not detected after the addition of AD or Chx to the suspension cell cultures (Figure 1E). Taken together, these data confirm that O_3_ induced a PCD in *A. thaliana* cells.

### Activation of anion channels is a crucial early event in O_3_-induced cell death

**Figure 2:**
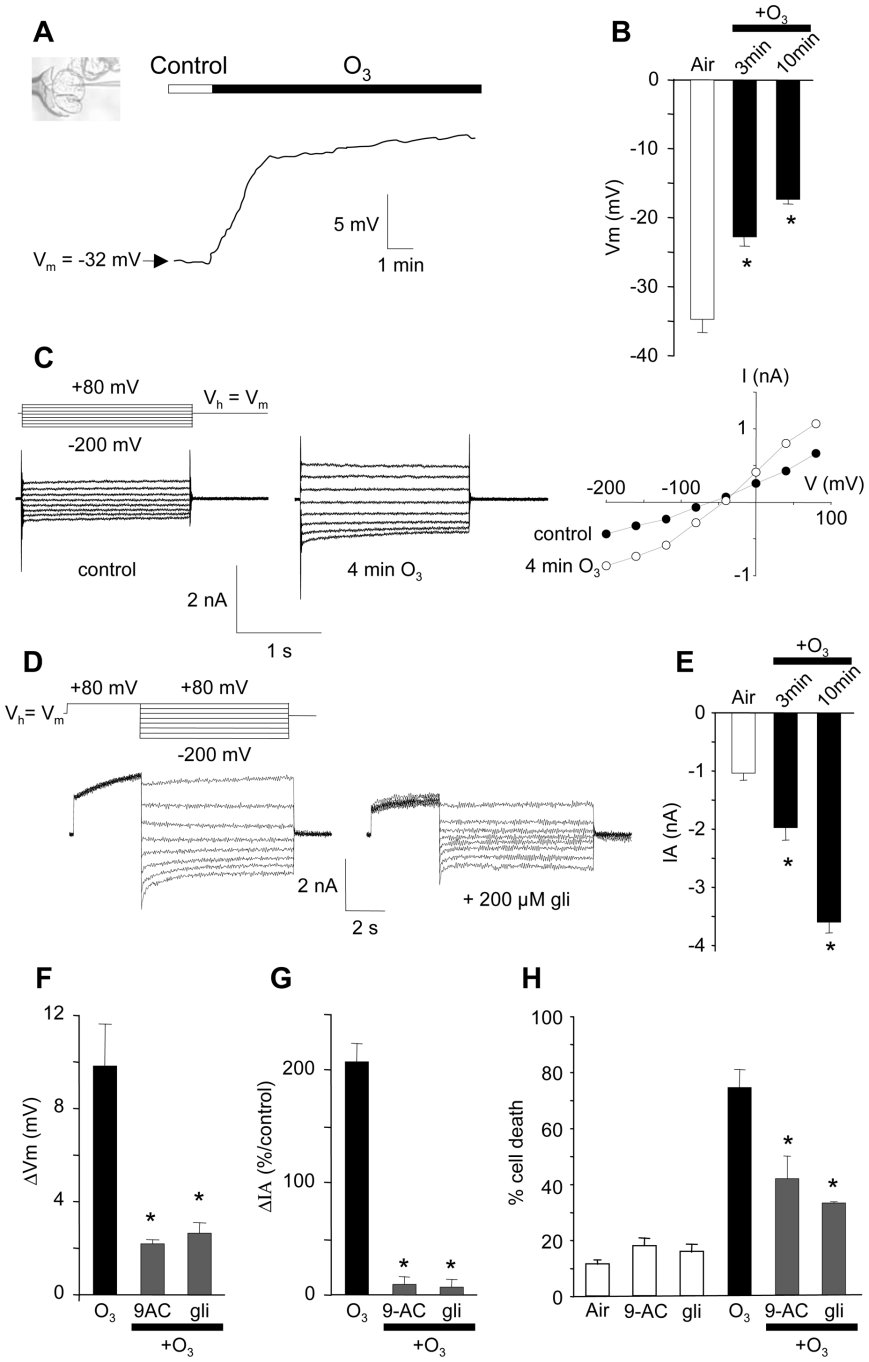
Ozone-induced depolarization and anion current increase of *A. thaliana* cells. **A.** Typical depolarization of an *Arabidopsis thaliana* cultured cell observed in response to O_3_ exposure. Inset, *A. thaliana* cell maintained by a microfunnel and impaled on a microelectrode. **B.** Mean values of plasma membrane (PM) potentials recorded after a pulse of ozonized air lasting 3 or 10 min or after a 10 min air pulse. **C.** Anion currents measured under control conditions and 4 min after O_3_ exposure. The protocol was as illustrated, holding potential (V_h_) was V_m_. Corresponding current-voltage relationships at 1.8 s. **D.** Anion currents showing slow activation, slow deactivation recorded after an ozone treatment. Decrease of current intensity by glibenclamide (200 µM) using the indicated protocols. **E.** Mean values of corresponding anion currents (recorded at −200 mV and 1.8 s) after the pulse of ozonized air lasting 3 or 10 min or after a 10 min air pulse. **F.** Mean values of depolarization recorded after 3 min exposure to O_3_ with or without anion channel blockers (200 µM 9-anthracen carboxylic acid (9-AC) or 200 µM glibenclamide (gli)). **G.** Mean steady state values of corresponding anion currents recorded at −200 mV and 1.8 s with or without 200 µM 9-AC in the medium. Current variations are given as a percentage of the control level before O_3_ exposure. Data correspond to mean values ± SD of at least six independent experiments. **H.** Effect of pretreatment with 9-AC or gli (200 µM each) on O_3_ induced-cell death. Cell death was induced by exposing the cells to ozonized air for 10 min. For development of cell death, cells were incubated for a further 6 h after O_3_ exposure. The data correspond to means of at least 4 independent replicates and error bars correspond to SE.

Cell shrinkage is a major hallmark of PCD. This process may be mediated by a net efflux of water resulting from the release of anions and K^+^. Indeed, anion efflux, detectable as a current increase, has been reported to be a necessary event to achieve cell death in suspension cells subjected to either cryptogein [33] or oxalic acid [37]. Since ozone induced cell shrinkage (Figure 1A), an electrophysiological approach was undertaken to determine the role of O_3_ on cell membrane potential and on anion currents. Two protocols were used to assess the O_3_ effect on cell polarization: (i) free-running PM potential time-courses were recorded in cells exposed to ozonized air (Figure 2A) and (ii) the mean PM potentials of cell populations exposed to ozonized air for 3 or 10 min were compared to those of cells pretreated by air alone during 10 min (Figure. 2B). The value of the resting membrane potential (Vm) of control cells (air treated or without treatment) were similar, −34.8±1.5 mV (n = 23) and −35,3±1.4 mV (n = 22) respectively and in the same range of previous studies [26], [41], [42], [43], [44]. A rapid depolarization of the cell PM in response to ozonized air was detected with the first protocol (Figure 2A). Accordingly, 3 or 10 min O_3_-exposed cells showed a depolarized PM compared to cells treated with air. The amplitude of the observed PM depolarization depended on the duration of the O_3_ treatment (Figure 2B). Previous electrophysiological studies and pharmacological analyses identified a current displaying the characteristics of anion channels in the PM of *A. thaliana* cells [24], [41]. This current was shown to be sensitive to structurally unrelated anion channel inhibitors, 9-anthracen carboxylic acid (9-AC) and glibenclamide (gli) [24], [42]. It presented features of slow anion channels [41], slow activation upon hyperpolarization and slow deactivation upon depolarization [45], although part of the instantaneous current could have been carried out by fast activating anion channels as described for guard cells [46]. Since long hyperpolarizing or depolarizing voltages could artificially modify the ionic content of our living cells, we recorded the signature of this current using shorter voltage pulses [41]. In accordance to the PM depolarization, O_3_ induced an increase of anion current after 4 min (Figure 2C). A current showing the features of slow anion channels and a sensitivity to glibenclamide was also detected when cells were exposed to O_3_ (Figure 2D). When compared to air treated cells, the anion current amplitude increase depended on O_3_ treatment duration (Figure 2E), reaching 200% of the mean control value of −1.17±0.05 nA (n = 21). The increase in anion current, that correlated to the PM depolarization amplitude (Figure 2B), might explain the depolarization induced by O_3_ since pretreatment of cells with gli or 9-AC (200 µM) drastically reduced the O_3_-induced depolarization and anion current increase (Figure 2F–G). Therefore, the effect of anion channel blockers on the extent of O_3_-induced cell death was tested. Ozone (10 min treatment) induced around 80% of cell death within 6 h (Figure 1B–C). When treated with anion channel blockers, gli or 9-AC (200 µM), cell death was reduced by approximately 50% (Figure 2H). These results suggested that the anion current increase was a required upstream event in the signaling pathway leading to O_3_-induced cell death.

### Ozone- induced calcium influx

**Figure 3:**
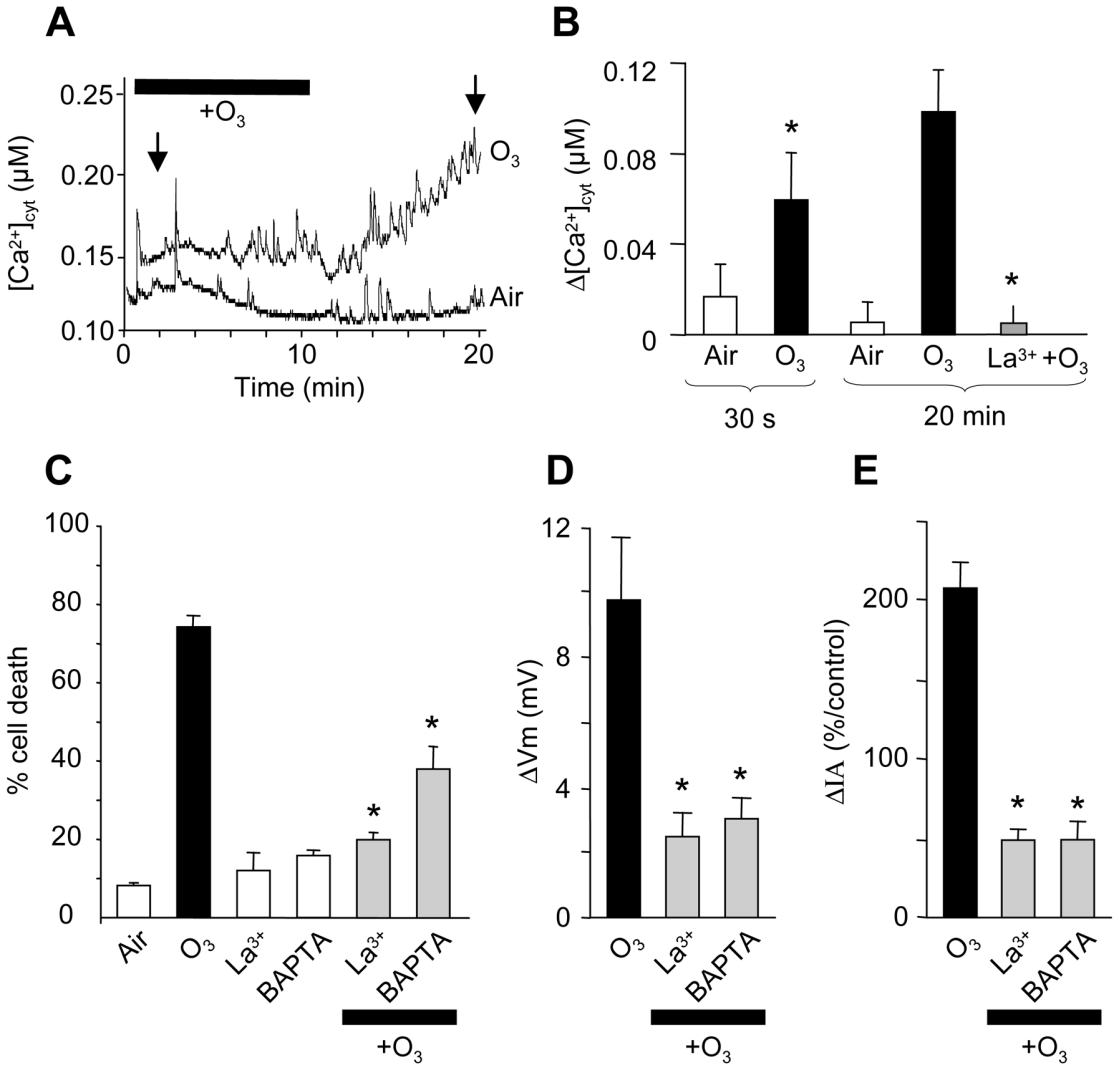
Ozone induced variations of Ca^2+^ in *A. thaliana* cells. **A.** A typical [Ca^2+^]_cyt_ variation of an aequorin expressing *Arabidopsis thaliana* cell in response to O_3_. **B.** Mean values of [Ca^2+^]_cyt_ variation after 30 s and 20 min (arrows in (A)) with or without La^3+^ (500 µM). **C.** Impact of BAPTA (1 mM) or La^3+^ (500 µM) on O_3_-induced cell death. Cell death was induced by exposing the cells to ozonized air for 10 min. For development of cell death, cells were incubated for a further 6 h after O_3_ exposure. Prior to O_3_ exposure, cell suspensions were treated with BAPTA (1 mM) or La^3+^ (500 µM). Evans blue stained cells were counted for each treatment. Data reflect the mean and SE of at least four independent experiments. **D.** Mean values of depolarization recorded after 3 min exposure to O_3_ with or without BAPTA or La^3+^. **E.** Mean steady state values of the corresponding anion currents recorded at −200 mV and 1.8 s with or without BAPTA or La^3+^. Current variations are given as a percentage of the control level before O_3_ exposure. Data correspond to mean values ± SD of at least six independent experiments.

Increases in [Ca^2+^]_cyt_ in response to O_3_ have been reported previously in Arabidopsis seedlings [4], [8], [9] and in tobacco cells [10]. Moreover, addition of Ca^2+^ chelators or Ca^2+^ channel blockers resulted in a significant inhibition of an O_3_-induced [Ca^2+^]_cyt_ increase and subsequent cell death suggesting that the uptake of extracellular Ca^2+^ via the activation of PM Ca^2+^ channels is required for the induction of active cell death [4], [10]. A change in [Ca^2+^]_cyt_ was detected in Arabidopis cells expressing aequorin in response to O_3_, as shown in Figure 3A. The observed changes in aequorin luminescence were biphasic, consisting of an immediate small increase in [Ca^2+^]_cyt_ immediately after the initiation of O_3_ exposure that was followed by a larger increase when O_3_ exposure was stopped (Figure 3A). This increase is maintained and reached a plateau after 50 min (data not shown). The Ca^2+^ channel blocker, La^3+^ (500 µM) inhibited the second phase of [Ca^2+^]_cyt_ increase after O_3_ exposure (Figure 3B), indicating that the influx of extracellular Ca^2+^ into the cells was via an activation of Ca^2+^ channels. Addition of 500 µM La^3+^ also resulted in the significant inhibition of O_3_-induced cell death (Figure 3C). Similarly, addition of 1 mM BAPTA, a membrane-impermeable Ca^2+^ chelator that is active at a physiological pH range, resulted in the inhibition of O_3_-induced cell death (Figure 3C). These results suggested that the uptake of extracellular Ca^2+^ was also required for cell death induction in *A. thaliana* cells. We thus tested the effect of BAPTA and La^3+^ on the O_3_-induced increase in anion current and the subsequent cell depolarization. Pretreatment with BAPTA or La^3+^ significantly lowered the O_3_-induced depolarization and increase in anion current (Figure 3D–E). These results suggested that an increases in [Ca^2+^]_cyt_ was also an early upstream event in the signaling pathway leading to O_3_-induced cell death in *A. thaliana* cells, and also suggested the involvement of the anion current increase in this signaling pathway.

### Effects of ROS scavengers

**Figure 4:**
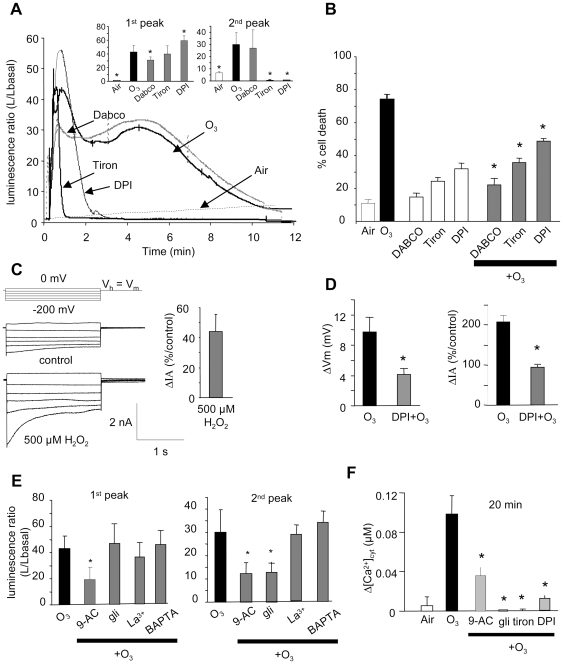
Ozone induced ROS generation in *A. thaliana* cells. **A.** Typical early biphasic ROS generation in *Arabidopsis thaliana* cells in response to O_3_ with or without DABCO (5 mM), tiron (5 mM), or DPI (50 µM). Inset showing the mean values of ROS generation for the first and 2^nd^ peaks.**B.** Cell death was induced by exposing cells to ozonized air for 10 min. For development of cell death, cells were incubated for a further 6 h after O_3_ exposure. Prior to O_3_ exposure, cell suspensions were treated with DABCO (5 mM), tiron (5 mM), or DPI (50 µM). Evans blue stained cells were counted for each treatment. Data reflects the mean and SE of 4 independent experiments. **C.** Anion currents measured under control conditions and 4 min after 500 µM H_2_O_2_ addition. The protocol was as illustrated, holding potential (V_h_) was V_m_. Mean current variations are given as a percentage of the control level before H_2_O_2_ addition. Data correspond to mean values ± SD of at least six independent experiments. **D.** Mean values of depolarization recorded after 3 min exposure to O_3_ with DPI 50 µM and mean steady state values of the corresponding anion currents recorded at −200 mV and 1.8 s with DPI. Current variations are given as a percentage of the control level before O_3_ exposure. **E.** Mean values of ROS generation for the 1st and 2^nd^ peaks in response to O_3_ with or without 9-AC or gli (200 µM each), La^3+^ (0.5 mM) or BAPTA (1 mM). **F.** Mean values of [Ca^2+^]_cyt_ variation after 20 min with or without 9-AC (200 µM), gli (200 µM), tiron (5 mM) or DPI (50 µM). Data correspond to mean values ± SE of at least six independent experiments.

The generation of ROS, such as superoxide anions (O_2_^ ˙−^) and hydrogen peroxide (H_2_O_2_) by O_3_ degradation in the apoplast, is a recognized mechanism involved in O_3_-induced damage [6], [11]. Addition of ROS scavengers or chelators was shown to inhibit O_3_ induced cell death, indicating that singlet oxygen (^1^O_2_), and H_2_O_2_ generated from O_2_^ ˙−^ by superoxide dismutase play central roles in acute O_3_-induced damage to tobacco cells [10]. We thus checked the effect of DABCO (a strong and selective scavenger of ^1^O_2_), tiron (a scavenger of O_2_^ ˙−^) and diphenyleneiodonium chloride (DPI, an inhibitor of the NADPH-oxidase), on O_3_-induced ROS generation. ROS generation was monitored by *Cypridina* luciferin analog (CLA) chemiluminescence which reports the presence of both O_2_^ ˙−^ and ^1^O_2_ (but to a lesser extent). Exposure of Arabidopis cells to O_3_ resulted in a biphasic enhancement of the CLA-chemiluminescence yield (Figure. 4A). An initial increase peaked at about 1 min while a second peak was observed at about 5 min. Since CLA is responsive to both O_2_^ ˙−^ and ^1^O_2_, tiron, DPI and DABCO were used to determine which ROS was involved [47]. Pretreament of the cells with DABCO (5 mM) allowed a significant decrease of the first peak while the 2nd peak remained unchanged (Figure 4A). Pretreatment of the cells with DPI (50 µM) or tiron (5 mM) failed to effect the first peak but drastically decreased the second peak of CLA-chemiluminescence (Figure 4A). Therefore, the first rapid chemiluminescence increase in the presence of CLA appeared to reflect the production of ^1^O_2_ (inhibited by DABCO), while a delayed production of O_2_^ ˙−^ (possibly converted to H_2_O_2_) is supported by the action of tiron and DPI (inhibition of the 2^nd^ peak). The effect of DABCO, tiron, and DPI on O_3_-induced *A. thaliana* cell death was also analysed after the cell suspensions were treated with DABCO (5 mM), tiron (5 mM) or DPI (50 µM) five minutes prior to O_3_ exposure. The O_3_-induced cell death was significantly reduced in the presence of DABCO, tiron and DPI (Figure 4B). Taken together, ROS generation is a likely component of the pathway leading to O_3_-dependent damage of *A. thaliana* cells, as previously suggested for tobacco and Arabidopsis seedlings [10], [48].

**Figure 5:**
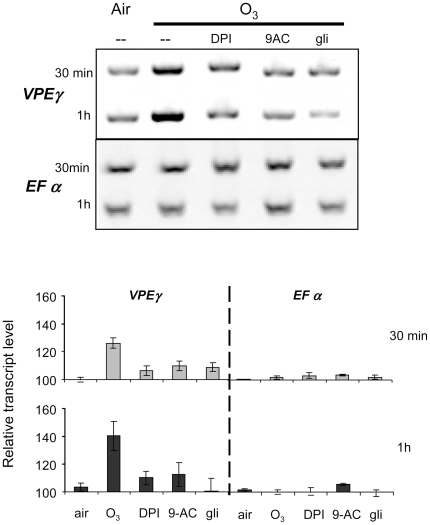
Effect of ozone on vacuolar processing enzyme gene transcription. The effect of a 10 min pulse of ozonized air on the transcription of a vacuolar processing enzyme (VPE) gene, encoding a caspase-like protein. Reverse transcriptase polymerase chain reaction (RT-PCR) was performed with RNA extracted 30 min and 1 h after the ozone pulse with or without DPI (50 µM), 9-AC or gli (200 µM each). EFαA4 was used as a housekeeping gene. Relative transcript level in different conditions. Quantitative evaluations were based on signal intensity analysed with ImageJ, and expression level of EFαA4 gene was used for calibration ( = 100). Results are means ± S.D. for three biological replicates.

Since (i) influx of Ca^2+^ could act upstream of the O_3_-induced activation of anion channels (Figure 4E) and (ii) H_2_O_2_ was shown to activate PM Ca^2+^ channels [49] and increase [Ca^2+^]_cyt_ in our model [25], we further tested whether H_2_O_2_ could activate anion channels. Indeed, application of 500 µM H_2_O_2_ induced an increase in anion current (Figure 4C) suggesting that O_3_-induced O_2_^ ˙−^ and subsequent H_2_O_2_ production could participate via Ca^2+^ influx to the O_3_-induced activation of anion channels. As expected from above data, pretreatment of cells with 50 µM DPI, thus blocking the O_3_-induced H_2_O_2_ generation (Figure 4A), significantly decreased the O_3_-induced depolarization and increase in anion channel activity (Figure 4D). The anion channel blockers gli and 9-AC (200 µM) were also found to be capable of decreasing the CLA luminescence increase observed upon O_3_ challenge (Figure 4E). Glibenclamide led to a decrease of only the second peak of O_3_-induced CLA-luminescence where as 9-AC decreased both peaks. Calcium channels blocker lanthanum or chelator BAPTA failed to decrease rapid ROS generation (Figure 4E). As a whole, these data suggested a complex interplay between anion channel regulation, Ca^2+^ influx and H_2_O_2_ production in response to O_3_. We thus checked if incubation, prior O_3_ exposure, with anion channel blokers (gli or 9-AC) or with the O_2_^ ˙−^ scavenger tiron and DPI could impact on O_3_-induced [Ca^2+^]_cyt_ increase. Effectively, anion channel blockers as H_2_O_2_ pharmacology (tiron and DPI) could reduced the late increase of cytosolic calcium (Figure 4F) confirming thus the interplay between anion channel regulation, Ca^2+^ influx and H_2_O_2_ production in response to O_3_.

### The O_3_-induced anion channel activation participates in vacuolar processing enzyme (VPE) gene expression

In mammalian cells undergoing AVD, the ion loss triggers activation of specific proteases [38]. Based on these data and on our finding that anion channel activity is involved in mediating vacuole shrinkage and O_3_-triggered cell death (Figure 1), we investigated whether the O_3_-induced anion efflux could be a key event in a signaling cascade leading to protease activation. Therefore, a putative role for the anion efflux in mediating the accumulation of mRNA encoding VPEs was investigated. This protease family has been shown to be essential for HR induction in tobacco challenged by pathogens [34], [50], and their transcription is dependent on anion efflux in response to cryptogein [33]. Four VPE genes have been identified in Arabidopsis, namely *VPEα*, *VPEβ*, *VPEδ*, and *VPEγ* [51]. To investigate the involvement of VPEs in O_3_-induced effects, we analyzed *VPE* mRNA accumulation in O_3_-treated cells by RT-PCR. Transcripts for *VPEα*, *VPEβ* and *VPEδ* were not detected in our model system (data not shown) however, the mRNA level of *VPEγ* increased in response to O_3_ with the changes rapidly occurring within 30 min (Figure 5) thus supporting the idea that VPEs participate in O_3_-induced damage in Arabidopsis. When Arabidopsis cells were treated with gli, 9-AC or DPI before the O_3_ challenge, accumulation of *VPEγ* transcript was reduced (Figure 5). Therefore, anion channel activation and H_2_O_2_ generation could be involved in the pathway leading to a transcriptional regulation of *VPEγ* transcripts.

### Hormones and anion channels in response to O_3_

**Figure 6:**
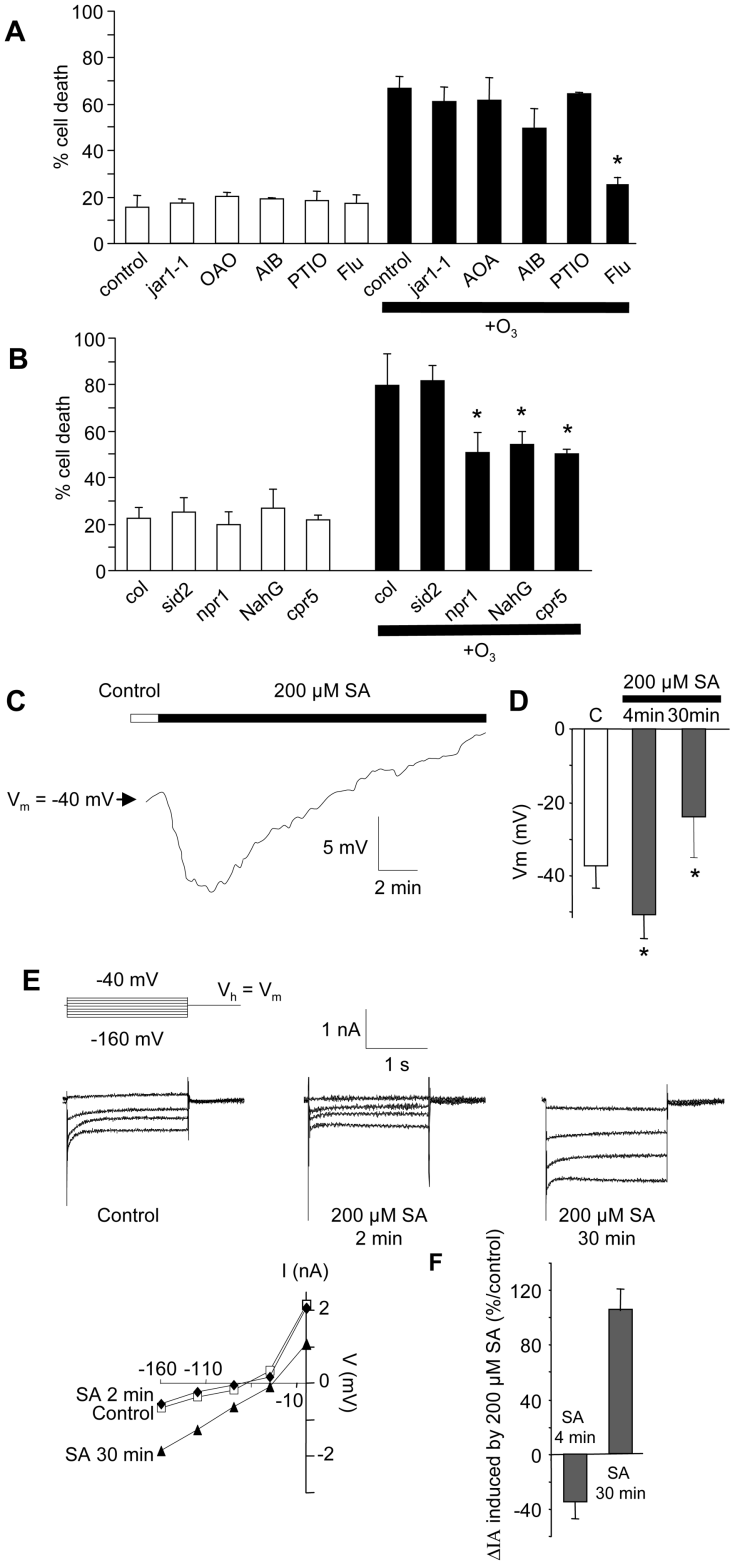
Role of hormone in O_3_-induced cell death in *A. thaliana* cells. **A.** Ozone induced cell death in suspension cells generated from jar1-1, or wild type cells pretreated with PTIO (250 µM), a scavenger of NO, with 200 µM of aminooxyacetic acid (AOA), an inhibitor of ACC synthase, with 200 µM of alpha-aminoisobutyric acid (AIB), an inhibitor of ACC oxidase, or with 100 µM fluoridon (Flu), an inhibitor of ABA synthesis. **B.** Ozone induced cell death in suspension cells generated from NahG, cpr5, sid2 and npr1 plants. Cell death was induced by exposing the cells to ozonized air for 10 min. For development of cell death, cells were incubated for a further 6 h after O_3_ exposure. The data correspond to means of at least 4 independent replicates and error bars correspond to SE. **C.** Time course of PM potential variation observed in response to 200 µM SA. **D.** Mean values of PM potential recorded 4 and 30 min after SA addition. **E.** Anion currents measured under control conditions, 4 and 30 min after addition of 200 µM SA. Protocols were as illustrated, holding potential (V_h_) was V_m_. Corresponding current-voltage relationships at 1.8 s. **F.** Mean current variations after SA addition given as a percentage of the control level before SA addition. Data correspond to mean values ± SD of at least six independent experiments.

Since the plant hormones SA, JA, ABA and ET as well as NO are involved in determining the duration and extent of O_3_-induced cell death and its propagation [3], [11], [19], [52], their impact was checked in our model system. Ozone-induced cell death was analysed in suspension cells generated from NahG, cpr5 and npr1 plants, which are impaired in SA signalling, the sid2 mutant, which is impaired in SA synthesis through the isochorismate pathway, and the JA-resistant mutant jar1-1. For ethylene, ABA and NO, pharmacological approaches were undertaken with (i) aminooxyacétique acid (AOA), an inhibitor of ACC synthase [53], and alpha-aminoisobutyric acid (AIB), an inhibitor of ACC oxidase [54] for ET, (ii) fluridon an inhibitor of ABA synthesis [55] and (iii) PTIO, a scavenger of NO [56]. Ozone-induced cell death levels recorded after pretreament of the cells with AOA, AIB and PTIO or with the jar1-1 cell line were not significantly different from that after O_3_ treatment alone in Col-0 background (Figure 6A), indicating that JA, ET and NO are not major actors in the signalling pathways leading to O_3_-induced cell death in Arabidopsis cultured cells. On the other hand, ABA synthesis appeared to take place in response to O_3_ since pretreatment of cells with fluridon counteracted the O_3_ effect (Figure 6A). In a similar manner, the O_3_-induced cell death extent in the NahG, cpr5 and npr1 cell lines showed significant decreases (Figure 6B), revealing that O_3_-induced death depended on SA signalling in Arabidopsis cultured cells. Interestingly, the sid2 cell line showed the same degree of O_3_ induced cell death as the wild-type Col-0 line (Figure 6B) suggesting that SA synthesis via the isochorismate pathway was not required for cell death induction in our cells.

As relatively high concentration of SA could be rapidly released in the apoplast from the storage form salicylic acid-glucoside (SAG) [57], [58], we tested the putative impact of SA on anion channel activation. Salycilic acid at 200 µM, a physiological concentration, induced a slight but rapid hyperpolarisation of the cells followed by a large depolarisation within a few minutes (Figure 6C–D). This biphasic regulation of the PM potential was accompanied by the biphasic regulation of anion channel activity, the delayed depolarization being correlated with an increased anion channel activity (Figure 6E–F). Thus, SA is not responsible for the early depolarization induced by O_3_ but it could participate to the anion channel-mediated depolarization in a delayed manner.

## Discussion

In this study we demonstrated that an acute exposure of Arabidopsis cells to ozone induced a controlled cell death displaying nuclear DNA fragmentation that required active gene expression and *de novo* protein synthesis. However, a typical laddering as previously reported in O_3_ treated tobacco leaves [5] was not observed. Ozone-induced cell death was only partially decrease by AD or Chx treatment whereas no DNA fragmentation was detected when cells were pretreated with these chemicals. Thus, we can not exclude that a small proportion of the cell death observed could be due to non active process. However, O_3_ also induced cell shrinkage, another hallmark of the PCD process in both plant and animal cells [35], [59]. These data fulfill the widely accepted criteria for PCD and confirm that our model responds to acute O_3_ exposure in the same way as previously described for other plants [3], [4], [5], [10].

We analyzed the putative role of anion channels in O_3_-induced cell death signalling pathways by using the microelectrode voltage clamp technique which allows working on living cells with their cell wall. An increase in anion current and a depolarization of the PM were observed after treatment of cell suspensions with O_3_. As previously discussed [24], [41], the anion currents we recorded showed the characteristic kinetic features of S-type anion channels responsible for long-term anion efflux and depolarization in guard cells [60]. The addition, before an O_3_ treatment, of 9-AC or glibenclamide, two structurally unrelated anion channel inhibitors was shown to be effective in *A. thaliana* suspension cells [37], [42], strongly reduced the anion current and partially prevented the O_3_–induced PM depolarization thus suggesting the participation of slow anion channel currents. Such a causal link has been described already with anion channel inhibitors which block blue light-induced depolarization [61] or toxin-induced depolarization [33], [44]. Activation of rapid- and/or S-type anion channels by ABA has been shown also to lead to the PM depolarization of guard cells in *Vicia faba* and *Arabidopsis thaliana* [60], [62]. In guard cells the long-term anion efflux and sustained depolarization has mainly been attributed to the activity of S-type anion channels. This, together with our finding that O_3_ induced anion currents displaying characteristic of slow type anion channels, suggests that the mechanism by which O_3_ promotes PM depolarization resembles that occurring in guard cells. It is noteworthy that recently, SLAC1, which represents the slow type anion channel of guard cells [28], [29], was shown to be essential for stomatal closure in response to O_3_, Ca^2+^ and H_2_O_2_ [31]. However, our data are the first direct evidence that O_3_ can regulate anion channel activity and that this anion current increase is an early prerequisite to the morphological and biochemical events participating to PCD. The activation of anion currents appeared to be important in the O_3_ response since 9-AC and gli decreased the induced cell death. Involvement of ion release via anion flux modulation is also considered to be essential among the earliest responses of plant cells to avirulent pathogens or elicitors capable of inducing PCD [63]. Our data pointed out a critical role for anion channels in the signaling pathways leading to cell death. In various mammalian cell types, AVD, which is mediated by water loss caused by activation of anion channels and a K^+^ outward rectifying channel, is an early prerequisite to apoptotic events including cell shrinkage, cytochrome c release, activation of proteases (including caspases) and nucleases, and ultimately PCD [39], [64]. Indeed, activation of outward K^+^ channels was reported in response to O_3_ in guard cell protoplasts [63], [65]. Thus, efflux of anions and K^+^ might drive water efflux leading to the observed cell shrinkage and death in response to O_3_.

**Figure 7:**
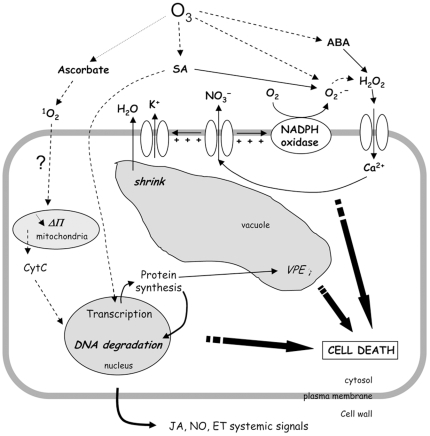
Hypothetical scheme for anion channel role in ozone signaling leading to cell death in *Arabidopsis thaliana* cells.

The involvement of ROS generation, increases in [Ca^2+^]_cyt_ and the crosstalk between these events in response to O_3_ is now widely accepted [3], [4], [8], [9], [10], [11]. Furthermore, variations in both ROS and [Ca^2+^]_cyt_ levels are involved in the regulation of anion channels in response to ABA in Arabidopsis cells [24], [25]. In our model system, although the first rapid increase in [Ca^2+^]_cyt_ did not resemble that reported in Arabidopis seedlings [9], the 2nd delayed increase was sensitive to La^3+^, thus suggesting that O_3_ induced an influx through PM Ca^2+^ channels [8], [9]. ROS generation was also detected in our experimental system. The impact of tiron and DPI strongly suggested that O_2_^ ˙−^ and subsequently H_2_O_2_ were produced by NADPH-oxidase in response to O_3_ as previously reported [16]. As expected from these data, a significant inhibition of O_3_-induced cell death was found after addition of BAPTA, La^3+^, tiron or DPI [4], [10]. These events appear to be linked to O_3_-induced anion channel increase activity since we observed that H_2_O_2_, which induced an increase in [Ca^2+^]_cyt_ in our model [25] probably through PM Ca^2+^ channel activation [49], was also capable of increasing anion channel activity. Accordingly, the O_2_^ ˙−^ scavenger tiron and NADPH-oxidase inhibitor DPI were able to decrease the late [Ca^2+^]_cyt_ variation. BAPTA and La^3+^ led to a decrease of the O_3_-induced depolarization and anion channel activation in accordance with the sensitivity of PM anion currents to an increase in [Ca^2+^]_cyt_ [24], [26] but have no effect on rapid ROS generation. Taken together, these data suggest that Ca^2+^ influx and ROS generation probably act upstream of anion channel regulation. However, the anion channel inhibitors gli and 9-AC were also shown to decrease O_3_-induced O_2_^ ˙−^ generation and [Ca^2+^]_cyt_ variation indicating a more complex interplay between ROS, Ca^2+^ and ion channel activation in signal transduction processes. A putative explanation might be that a first non biological ROS generation, from O_3_ reacting with the ascorbate pool [6], induced the Ca^2+^ influx required for the activation of an anion channel. In turn, the Ca^2+^-activated anion channel and the ensuing PM depolarization possibly amplify the O_3_ signal by activating a PM NADPH-oxidase as recently described in animal cells [66], [67]. The H_2_O_2_ derived from the NADPH-oxidase activity then participates in the increase of [Ca^2+^]_cyt_, through the activation of PM Ca^2+^ channels [9], [25], [49] acting as a feedback loop on anion channel activation. This scenario is reminiscent of the activation of the oxidative burst in aequorin-transformed *Nicotiana tabacum* cells which was shown to be mediated by an anion channel-dependent increase in [Ca^2+^]_cyt_ [68], [69]. However it is to be note that this “wheel” of interplay (Figure 7) might be fuelled by several other entries such salycilic acid which could also fuel the generation of H_2_O_2_ and Ca^2+^ influx involved in O_3_-induced cell death. SA-induced generation of ROS by cell wall peroxidases [70], [71] and by NADPH-oxidase [58] is effectively known to lead to the Ca^2+^ influx, and this could explain the delayed increase in anion current observed in response to the application of exogenous SA. Active SA could be released from the inactive SAG pool in apoplast through SAGase action [58], [72]. This could explain why the extent of cell death could be minimized after an O_3_ challenge in SA-related signalling cell lines (NahG, cpr5 and npr1), while that cell death level in the sid2 mutant impaired in SA synthesis could not be. Our data clearly confirm that SA is involved in the amplification of O_3_-induced cell death [4], [16]. In the same way, ABA synthesis could also fuel this mechanism since this hormone is known to induce ROS generation, cytosolic Ca^2+^ increases and anion channel increases in Arabidopsis cells [25]. The impact of ABA on ion fluxes in response to O_3_ is reminiscent of the ABA effect on stomata allowing sustained ion efflux leading to a decrease in cell volume. This is in accordance with the recent observation that SLAC1 is essential for stomatal closure in response to ozone, ABA, Ca^2+^ ions, and H_2_O_2_ in Arabidopsis [31]. Thus, in our model SA release and ABA synthesis induced by O_3_ could participate to sustain PM depolarization and anion effluxes leading to cell shrinkage.

The mitochondrial pathway was shown to be implicated in O_3_-triggered PCD [5]. However, it did not appear to be linked to anion channel activation since glibenclamide which decreased the activation of O_3_-induced anion channels, thus leading to less cell death failed to stop the O_3_-induced Δψm (Figure S1). Nevertheless, DABCO, a ^1^O_2_ scavenger which decreased the ^1^O_2_ level and cell death in our model, inhibited the effect of O_3_ on Δψm (Figure S1). The reaction of O_3_ with ascorbate is known to lead to high yields of singlet oxygen [6] and therefore the detection of a rapid ^1^O_2_ production in response to O_3_ was not a surprise. However, the involvement of ^1^O_2_ in O_3_-induced cell death might not involve the same pathway as that induced by anion channels, thus reinforcing the idea that several different pathways leading to cell death are triggered in response to O_3_. Further studies are needed to understand the role of ^1^O_2_ in O_3_-induced cell death.

To further assess the mechanisms by which the anion current increase contributes to cell death, its participation in the transcriptional activation of VPEs identified as key players in plant PCD [73] was explored. VPE is a family of cysteine proteases which exhibit enzymatic properties similar to that of caspase-1, a cysteine protease involved in the PCD pathway in animals. Our study was based on the finding that O_3_ induces protease activities [4], [5] and that VPE transcript levels are dependent on anion channel mediated NO_3_^−^ effluxes during cryptogein-induced cell death in tobacco [33]. Our results show that O_3_ treatment up-regulates the expression of *VPEγ* and not the other Arabidopsis VPE-encoding genes. In the presence of 9-AC, glibenclamide or DPI, the accumulation of *VPEγ* transcripts was reduced. The activation of O_3_-induced NADPH-oxidase and anion channels could thus be early prerequisites for *VPEγ* synthesis. Our data are in accordance with the fact that VPE activities can be increased by SA treatment and that expression of *VPEγ* is also transiently up-regulated during the early phase of HR activation [35], [74], at judged by increased transcript levels. Although the VPE target(s) that mediates HR cell death is unknown, Hatsugai et al. [34] nevertheless observed a dramatic inhibition of vacuole collapse in VPE-suppressed plants. Thus a VPE-dependent disruption of vacuole integrity might be a crucial step in O_3_-induced vacuolar shrinkage and cell death, as it is for some forms of HR cell death [33], [34], [73]. Therefore, anion channel activation might not be a passive secondary aspect of O_3_-induced cell death, but an event that drives the whole process.

Plant hormones as well as NO are involved in determining the duration and extent of O_3_-induced cell death and its propagation [3], [11], [19], [52]. The extent of O_3_-induced cell death with the JA-resistant mutant jar1-1 and cells treated with pharmacological agents that block ethylene or NO synthesis was similar to that observed in the wild type cell line. This suggested that in Arabidopsis suspension cells, JA, ET and NO were not involved in the development of O_3_-induced PCD. It should be noted that methyl-jasmonate, jasmonic acid, diethylamineNONOate, used as an NO donor [56], or the ET generator aminocyclopropane carboxylate failed to increase anion channel activity under our experimental conditions (data not shown). However, our data are not in opposition with the involvement of JA and ET in determining the duration and extent of O_3_-induced cell death and its propagation [3]. In the same way, NO might be needed to generate the intracellular signals required for the cell-to-cell spreading of an O_3_-induced HR, but not necessary to induce a PCD [19]. In our experiments all of the cells were challenged by O_3_, thus systemic messages could not impact on the degree of cell death. Our data are thus in accordance with a direct role for SA and ABA in O_3_-induced cell death while JA, ET and NO probably act only in auxiliary signalling pathways, which stabilize and amplify the primary signal without excluding a role for SA and ABA in these auxiliary signalling pathways [3], [11], [75].

In summary, this work shows that anion channel activation is central to the signalling cascade leading to O_3_ induced-cell death and provides evidence that anion movements are tightly correlated to the cellular and molecular events involved in this process. Indeed, anion channels are now recognized as important players in signaling pathways associated with plant cell responses to abiotic and biotic environmental stresses [21], [22] and our findings highlight the notion that plants, like animals, use anion channels as components of cell death pathways.

## Materials and Methods

### Cell culture conditions

For this study, *Arabidopsis thaliana* L. cell line T87 [76] was used. Axelos et al. (1992) [76] have previously established a cell line (named T87) from the ecotype Columbia plant. Suspension cells have been shown to be a convenient model for identifying early physiological events induced by different biotic [32], [37], [42] and abiotic stress [10], [77]. They show physiological responses to various stimuli, in a similar manner to autonomous cellular responses in intact tissues [78], especially the morphological features of dying cells during PCD [79], and thus allow the observation of events in each single cell or the real time behavioral monitoring of large populations of cells. *A. thaliana* suspension cells were grown in Gamborg or Murashige-Skoog medium (pH 5.8). They were maintained at 22±2°C, under continuous white light (40 µE m^−2^ s^−1^) and continuous shaking (gyratory shaker) at 120 rpm. Cell suspensions were sub-cultured weekly using a 1∶10 dilution. All experiments were performed at 22±2°C using log-phase cells (4 days after sub-culture).

### Preparation of Arabidopsis mutant or transgenic cell lines

For the cell suspension cultures derived from Arabidopsis mutants (jar1-1, sid2-1, cpr5 and npr1) and transgenic lines (NahG and apoaequorin), the seeds of mutants and transgenic lines were sterilized in 1% (w/v) sodium hypochlorite and allowed to germinate on sterilized MS agar plates containing vitamin B5, but lacking 2,4-dichlorophenoxy acetic acid (2,4-D). The seedlings were grown on the agar plates under a 12/12 h light/dark regime at 23±1°C for three weeks. Excised tissues from harvested seedlings were transferred onto agar medium containing 0.2 mg/ml 2,4-D to promote callus formation. Suspension cultures of cells were initiated by addition of cut pieces of the resulting calli to the MS or Gamborg liquid medium (pH 5.8) containing 0.2 mg/ml 2,4-D. The cell suspension cultures (30 ml each in 100 ml conical flasks) were kept on gyratory shakers (120 rpm) at 22±2°C under continuous light, and sub-cultured using 30 ml of 7-day cultures as inocula. Cells were harvested for the O_3_ experiments 4-days after sub-culturing.

### Ozone exposure

Ozone exposure of the cell suspension was performed as previously described [10]. Ozonized air (0.1 L/min; 10 mg O_3_/h) was passed on the surface of the cell suspensions (250 µL in 4 mL tubes). By this way the cells could be exposed to the pulse of O_3_ for 3 or 10 min. Ozone was generated by a ceramic ozonizer (NAVI Super Ceramics Ozonizer EO-mini, Kenis Kagaku Kyoeisha Ltd., Tokyo, Japan), equipped with an air pump.

### Cell viability assays

Cell viability was assayed using the vital dye, Evans blue, after air or ozone treatment with or without the appropriate pharmacological effectors (pretreatment of 15 min prior O_3_ exposure). Cells (50 µl) were incubated for 5 min in 1 ml phosphate buffer pH 7 supplemented with Evans blue to a final concentration of 0.005%. Cells that accumulated Evans blue were considered dead. At least 1000 cells were counted for each independent treatment and repeated at least 4 times for each condition.

Cell viability was also checked using fluorescein diacetate (FDA) as previously described [43]. Briefly, after the appropriate treatment, 1 mL of cell suspension was gently stirred with a magnetic stirrer before FDA was added to a final concentration of 12 µM. The fluorescence increase was monitored over a 120 s period using a F-2000 spectrofluorimeter (Hitachi, Japan). Results are presented as the percentage of cell death =  (slope of treated cells/slope of non treated cells) * 100±SE. The experiment was repeated at least 4 times for each condition.

### DNA extraction and analysis

Frozen cells were ground in liquid nitrogen and genomic DNA was extracted according to the CTAB method [80]. DNA electrophoresis was performed to assess DNA fragmentation. DNA samples (5 µg/lane) were loaded onto a 1.8% agarose gel including 0.2 µg/ml ethidium bromide.

### Electrophysiology

Individual cells were impaled and voltage-clamped in the culture medium using an Axoclamp 2B amplifier (Axon Instruments, Foster City, CA, USA) for discontinuous single electrode voltage clamp experiments as previously described [26], [37], [43]. Voltage and current were digitized using a computer fitted with a Digidata 1320A acquisition board (Axon Instruments). The electrometer was driven by pClamp software (pCLAMP8, Axon Instruments). Experiments were conducted on 4-day-old cultures at 22±2°C (main ions in the medium after 4 d of culture: 9 mM K^+^, 11 mM NO_3_^−^, [41]).

### Aequorin luminescence measurements

Cytoplasmic Ca^2+^ variations were recorded using freshly generated *A. thaliana* cell suspensions (T87) expressing apoaequorin [81]. For calcium measurements, aequorin was reconstituted by the overnight incubation of the cell suspension in Gamborg medium containing 2.5 µM native coelenterazine. Cell culture aliquots (500 µL) were transferred carefully to a luminometer glass tube, and the luminescence counts were recorded continuously at 0.2 s intervals with a FB12-Berthold luminometer. Treatments with air or ozone were performed directly in the luminometer. At the end of each experiment, the residual aequorin was discharged by addition of 500 µL of a 1 M CaCl_2_ solution dissolved in 100% methanol. The resulting luminescence was used to estimate the total amount of aequorin for each condition. Calibration of calcium levels was performed using the equation: pCa =  0.332588(-logk)+5.5593, where k is a rate constant equal to luminescence counts per second divided by total remaining counts. Data are expressed as µM and are means ± SE.

### Monitoring of ROS Production

The production of singlet oxygen (^1^O_2_) and O_2_^ ˙−^ was monitored by the chemiluminescence of the *Cypridina* luciferin analog (CLA) as previously described [13]. CLA-chemiluminescence specifically indicates the presence of O_2_^ ˙−^, and of ^1^O_2_ to a lesser extent, but not other ROS [82]. Chemiluminescence from CLA was monitored using a FB12-Berthold luminometer (with a signal integrating time of 0.2 s). For the statistical analysis of the data, the luminescence ratio (L/Lbasal) was calculated by dividing the luminescence intensities of CLA-luminescence (L) with the luminescence intensity before air or ozone treatment (Lbasal).

### RT-PCR analysis of gene expression

Four-day-old cells were treated with O_3_, harvested and frozen in liquid nitrogen. Total RNA was prepared using the Genelute™ Mammalian Total RNA Kit (Sigma). RNA was treated by the Deoxyribonuclease I Kit (Sigma). Total RNA was quantified by spectrophotometry and their integrity checked on denaturing agarose gels. Total RNA (2 µg) was converted into first-strand cDNA with the Superscript™ II Rnase H^−^ Reverse Transcriptase Kit (Invitrogen, Carlsbad, CA, USA) and oligo(dT) primers. One µl of cDNA was amplified in a 25 µl PCR mixture. *VPEα*, *VPEβ*, and *VPEγ* primers were designed (*VPEα* forw: GCGAAGAACGAGGAGAATCCAA, *VPEα* rev: TGCTCGTGCAAAGTCTCTGTTT; *VPEβ* forw: ACAATGACCACCGTCGTTTCCT, *VPEβ* rev: TAGGGCGGAGACGAAGATCAAG; *VPEγ* forw: GACCATGGTGGTCCTGGAGTTC, *VPEγ* rev: ATTCGCATCCGCAAAGGTAAAA). Control PCR was performed using the housekeeping gene *EF1α4* [83]. Thermal cycling conditions were as follows: an initial denaturation step at 94°C 2 min, followed by 34 cycles (or by 26 cycles for *EF1α4*), of 94°C 30 s, 55°C 30 s, 72°C 1 min 30 s, and ending with a single step at 72°C 10 min. PCR products were seperated by gel electrophoresis and visualized by ethidium bromide fluorescence. Representative results from three independent experiments are shown.

## Supporting Information

Figure S1 **Effect of O_3_ on mitochondrial membrane potential (Δψm) of** ***A. thaliana*** **cells.** Mean values of JC-1 fluorescence ratio (high ψm versus low ψm) measured 15 minutes after exposing the cells to ozonized air for 10 min and effect of 5 mM dabco and 200 µM glibenclamide (gli) on the decrease of JC1 fluorescence ratio induced by O3. Valinomycin at 1 µM was used as a positive control. Data are representative of at least 4 independent experiments and error bars correspond to standard errors.
(0.10 MB TIF)
