# Supplementary figures and images for: Increased Anion Channel Activity Is an Unavoidable Event in Ozone-Induced Programmed Cell Death
Source: PLoS One. 2010 Oct 13;5(10):e13373. doi: 10.1371/journal.pone.0013373 (PMC2954175; doi:10.1371/journal.pone.0013373)

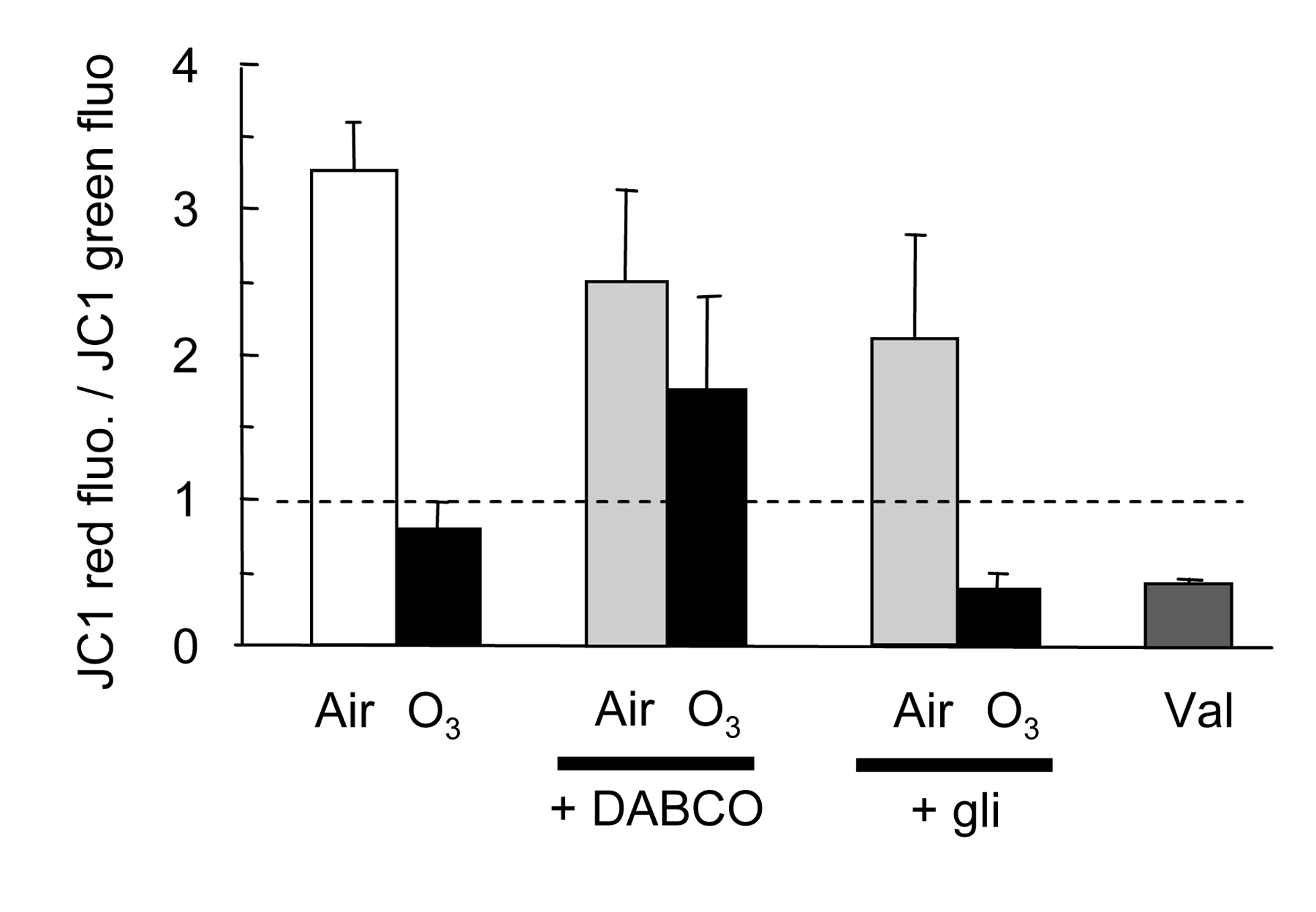

Supplement: Figure S1 — Effect of O3 on mitochondrial membrane potential (Δψm) of A. thaliana cells. Mean values of JC-1 fluorescence ratio (high ψm versus low ψm) measured 15 minutes after exposing the cells to ozonized air for 10 min and effect of 5 mM dabco and 200 µM glibenclamide (gli) on the decrease of JC1 fluorescence ratio induced by O3. Valinomycin at 1 µM was used as a positive control. Data are representative of at least 4 independent experiments and error bars correspond to standard errors. (0.10 MB TIF) [file pone.0013373.s001.tif]
